# A Literature Review of EEG-Based Affective Computing in Marketing

**DOI:** 10.3389/fpsyg.2021.602843

**Published:** 2021-03-16

**Authors:** Guanxiong Pei, Taihao Li

**Affiliations:** Zhejiang Laboratory, Research Center for Advanced AI Theory, Hangzhou, China

**Keywords:** electroencephalography, affective computing, marketing, neural affective mechanisms, classification and recognition

## Abstract

Affect plays an important role in the consumer decision-making process and there is growing interest in the development of new technologies and computational approaches that can interpret and recognize the affects of consumers, with benefits for marketing described in relation to both academia and industry. From an interdisciplinary perspective, this paper aims to review past studies focused on electroencephalography (EEG)-based affective computing (AC) in marketing, which provides a promising avenue for studying the mechanisms underlying affective states and developing recognition computational models to predict the psychological responses of customers. This review offers an introduction to EEG technology and an overview of EEG-based AC; provides a snapshot of the current state of the literature. It briefly presents the themes, challenges, and trends in studies of affect evaluation, recognition, and classification; and further proposes potential guidelines for researchers and marketers.

## Introduction

Affective computing (AC) is a continuously growing interdisciplinary research field spanning psychology, computer science, cognitive science, neuroscience, and more (Tao and Tan, 2005). In Picard’s landmark book, AC is defined as “computing that relates to, arises from, or deliberately influences emotion or other affective phenomena” (Picard, 1997). It mainly focuses on how technology can inform and deepen understanding of human affect and how systems can be designed to estimate the affective state using computational models from behavioral and physiological signals (Calvo and D’Mello, 2010).

Affect plays an important role in human cognition, specifically in perception processes, rational decision-making, human communication, and human intelligence (Ammar et al., 2010; Singh et al., 2013). In marketing, understanding and recognizing the affective states of users (or customers) has become a vital theme, which can reveal users’ true preferences and improve and assist in the purchasing process (Malär et al., 2011; Garrido-Morgado et al., 2015).

Conventional assessment methods of AC in marketing depend mostly on personal evaluations, such as surveys, focus groups, and interviews (Ariely and Berns, 2010; Yadava et al., 2017; Lin et al., 2018). However, customers may not express their true opinions because of social desirability bias (Paulhus, 2002; Vecchiato et al., 2011a). They may not say exactly how they are feeling but rather, how they feel others would reply (Calvert and Brammer, 2012). These *post hoc* analysis tools are also influenced by individuals’ mental states or environments at the time of self-reporting (Nilashi et al., 2020). Due to the limitations of traditional AC techniques, marketers and researchers try to supplement these shortcomings and seek alternative or complementary tools. Neuroscientific tools based on electrophysiological and neuroimaging techniques provide one such alternative, as well as a way to dig deeper into understanding the complex evaluation process and the dynamics of the affective state by directly accessing the physiological signals and fundamental cerebral structure from which an affective state occurs.

Considering its low cost and high temporal resolution (milliseconds), electroencephalography (EEG) has become common and is extensively used in the marketing industry. Furthermore, variations in EEG signals cannot be voluntarily controlled and therefore are a better objective indicator of affect (Singh et al., 2013). EEG-based AC provides a promising avenue for studying the mechanisms underlying affective states and developing recognition computational models to predict the psychological responses of customers. It can, therefore, be widely used to boost sales, advertising, pricing, package design, marketing campaigns, and so forth (Calvo and D’Mello, 2010).

In this review, we focus on EEG-based AC in marketing. First, we stress the need to incorporate the various features of neural signals that contribute to consumers’ affective states and evaluation processes beyond what traditional marketing measures already provide. Second, we state that marketing studies should adopt the methodologies and algorithms used in data processing and prediction modeling that are mature in other fields such as computer science and engineering. We examine AC literature in marketing on the general features extracted from EEG recordings and conclude with a general discussion of the challenges faced by this field, providing several recommended guidelines for the road ahead.

## Consumers’ Neural Affective Mechanisms

It is expected that a deeper understanding of how the human brain works and responds will make marketing more effective (Kuan et al., 2014; Nagyová et al., 2014; Caratù et al., 2018). Researchers and marketers have attempted to reveal the neural correlates and neural affective mechanisms that affect decision making in customers and consumer behavior in economic processes.

### Event-Related Potentials

As an extremely useful tool for investigating higher-level brain functions, event-related potentials (ERPs) have been widely used to explore the neural mechanisms underlying affect processing in marketing. For example, Ma et al. (2007) found that brand extension preferences were modulated by negative emotions. These underlying mechanisms were indicated by the amplitudes of N270. This research presents a method, which is automatic, objective, and non-verbal, for the use of ERP components as markers for emotional preferences. Jones et al. (2012) shifted their attention toward pricing and discount-related consumer behavior. The enhanced P3 in high math anxiety individuals indicated greater reliance on the emotional and motivational factors involved in the buying process. Social interaction in marketing is another important theme. In a study by Pozharliev et al. (2015), the late positive potential (LPP) was enlarged during shopping for luxury products. It was especially prominent in the presence of another person during buying decisions, suggesting that social interaction magnifies the emotional effect of the brand category. Furthermore, Chen et al. (2010) found that N500 was evoked by emotional conflicts when making counter conformity choices in buying books online.

By taking full advantage of the high temporal resolution of ERPs, a group of scientists conducted multistage experiments and multicomponent detections to decompose the time course of neural cognitive and affective activities. Handy et al. (2010) focused on the emotional appraisal of unfamiliar commercial logos. Using an ERP examination, they found that the judgment of logos can be divided into at least two stages: an initial formation of ones’ impression at the sensory-perceptual level and evaluative analysis at the cognitive-processing level. Jin et al. (2018) demonstrated that eco-labeling induced positive emotions in consumers and reduced cognitive conflict, as reflected by decreased P2 and N2 amplitudes, respectively. In another study, Shang et al. (2020) studied online shopping and focused on the influence of webpage layouts on consumer experience. They demonstrated that a low-order online shopping webpage facilitated consumers’ instant purchase decisions as indexed by increased P2 (attention engagement) and LPP (emotional self-control) amplitudes. Separating affective activities from cognitive activities and capturing independent processes within each stage is helpful to provide a deeper and more nuanced understanding of consumers’ neural affective mechanisms.

### EEG Time-Frequency Components

Because EEG signals comprise abundant time-frequency information, multiple characteristic parameters, such as hemispheric asymmetry, event-related desynchronization/synchronization (ERD/ERS), and power spectral density (PSD), are closely related to affective states.

Concerned with the frontal EEG asymmetry, Vecchiato et al. (2010) revealed that the cerebral activity of the theta band on the left frontal hemisphere increased when television commercials were judged to be pleasant. As a follow-up to this, Vecchiato et al. (2014) further highlighted that the index of cortical hemispheric asymmetry, which was also a valid predictor of preferences, was significantly correlated with participants’ pleasantness ratings. Furthermore, the frontal alpha band, as reflected in cerebral hemispheric alpha asymmetry, has been widely used to represent affective responses to advertisements (Ohme et al., 2010; Venkatraman et al., 2015). When two subjects were viewing two versions of the almost same skincare product advertisement (differing only by the inclusion of a very short clip concerning a gesture by a female model), Ohme et al. (2009) found out that each version elicited significantly different emotional impacts, indexed by differences in the frontal alpha activity between the left and the right hemispheres. Similarly, Reeves et al. (1989) reported a significant interaction between hemisphere activity and the emotional content (positive and negative) of television advertisements for frontal alpha power but no interaction for occipital alpha power. It would be more beneficial for future research to specify the role of mediation and moderation that frontal alpha asymmetry plays in relation to advertisements and affective states (Palmiero and Piccardi, 2017; Allen et al., 2018).

With regard to the ERD/ERS indices, Vecchiato et al. (2011b) demonstrated that desynchronization of the left alpha band is positively related to judgments of high pleasantness. Other findings also supported the evidence that synchronization and desynchronization changes were the result of affective arousal and valence (Aftanas et al., 2001; Costa et al., 2006). Moreover, ERD/ERS is a useful tool for understanding variation in oscillatory activity occurring during the decision-making process related to subjective preferences. Khushaba et al. (2013) explored brain activity while subjects were choosing crackers using EEG and eye tracking data. They found clear phase synchronization between the left and right frontal and occipital regions during preference decision making for the different cracker characteristics, which can be used to predict consumers’ future choices and develop effective marketing strategies.

By performing power spectra and PSD analyses, Horska et al. (2016) and Berčík et al. (2016a) studied the emotions of consumers tasting different kinds of wine and used their findings to improve the practical selling strategies. Subsequently, Berčík et al. (2016b) conducted interdisciplinary research on the impact of illumination on the emotional state (valence) of customers in a food store by calculating and comparing alpha and beta spectral power. Lee (2016) explored the emotional mechanism of empathy indexed by the theta-band power spectra of the anterior cingulate cortex (ACC) for customer equity and willingness to pay. Moreover, Vecchiato et al. (2011b) showed that an increase in PSD in the left frontal lobe is negatively related to the degree of perceived pleasantness. These studies indicate that power spectra and PSD are reliable indicators in identifying emotional responses to marketing.

Taken together, the studies discussed above provide evidence that it is helpful to use EEG features and properties to understand neural affective mechanisms, which enables us to objectively reveal hidden consumer reactions and identify critical success factors in the process of buying and consuming certain products (Kuan et al., 2014; Telpaz et al., 2015). We believe that brain wave-based methodologies would further enrich marketing research and help marketers to go beyond traditional marketing paradigms.

## Classification and Recognition of Affective States

Feature extraction is a critical step for obtaining an accurate classification result. It is beneficial for simplifying the models and the amount of resources, reducing the cost of data processing, improving data visualization, and avoiding overfitting (Übeyli, 2008; Al-Fahoum et al., 2014). Various methods have been used to extract the neural features from EEG signals. Among these methods are time frequency distributions (appropriate for great continuous segments), fast Fourier transform (suitable for narrowband signal and stationary signal), wavelet transform (good tool for sudden and transient signal changes), auto regressive methods (which are advantageous for short data segments), and so on (Tao and Tan, 2005; Singh et al., 2013; Al-Fahoum et al., 2014; Yadava et al., 2017). Researchers should make clear the signal types and apply the optimum method.

Once the features are extracted, the features will be fed into an affect classification algorithm. Many types of classifiers are commonly used including: support vector machines (SVMs), relevance vector machines (RVMs), logistic model trees (LMTs), Fisher linear discriminant analysis (FLDA), the k-nearest neighbors (KNN), hidden Markov models (HMMs), artificial neural networks (ANNs), artificial bee colonies (ABCs), random forests (RFs), and deep neural networks (DNNs). In the following paragraphs, we present the various types of affective classifiers used in marketing scenarios.

Many studies have used machine learning algorithms to assess the impact of advertising. Friedman et al. (2015) proposed an EEG data-driven approach to measure customers’ emotional valence when processing commercials. Their results indicated that hemispheric asymmetry was a good marker and the LMT algorithm (81.2%) provided better classification rates than the SVM algorithm (77.3%). Wei et al. (2018) explored a new method using low-cost EEG headbands to assess the influence of advertisements on purchasing. The EEG features that were closely related to emotions were gathered into different groups and the SVM method was applied to assess the ability of features to predict possible purchases, achieving an accuracy of 77.3%. Yang et al. (2015) developed an approach to evaluate the temporal patterns of EEG data (PSD) and extract affective indices such as happiness and surprise for TV commercial evaluation. FLDA was used to predict which parts of an advertisement could elicit positive emotional responses in customers. Similarly, Guixeres et al. (2017) attempted to forecast the effectiveness of advertisements during the Super Bowl sports event in the United States, based on EEG signals, including biometric responses such as the z-score of the global field power (GFP), hemispheric asymmetry (pleasantness index), and the relative number of peaks in the beta and theta bands (interest index). The results showed that the ANN was able to precisely classify and estimate the effect of each advertisement on the Internet *via* biometrics (82.9% of average accuracy). Marketers could consider the proposed approach at the technical design stages of advertising content. From a multimodal perspective, Gauba et al. (2017) developed a notable rating forecast framework for advertisement clips by using both EEG signals and sentiment analysis of online users’ comments from YouTube. The prediction was carried out using the RF regression and was later fused with the sentiment score to improve the overall prediction.

Some scholars pay special attention to the music or jingles of advertisements. Lin et al. (2014) focused on the emotion classification problem when listening to music and used the SVM classifier, generating accuracies of 82.5 and 79.1% for valence and arousal classification, respectively. Gupta and Falk (2016) used EEG graph-theoretic features to classify emotional states while watching music clips with the aid of two classifiers, the SVM and RVM, and the approach achieved a significant improvement in the classification accuracy (The percentage increase in classification performance ranged from 3 to 9%). More recently, Avinash et al. (2018) developed a very accurate tool for understanding consumers’ emotional responses to advertisement jingles. The KNN algorithm was used to classify positive and negative emotions based on theta power signals, achieving an accuracy of 100%.

Other studies have investigated consumers’ purchasing preferences. Chew et al. (2016) studied the aesthetic emotional responses in industrial design and buying decisions using EEG signals for virtual 3D shapes with motion. A classification accuracy of up to 80% was attained using the KNN with the alpha, theta, and delta rhythms as the features taken from frontal electrodes to classify two classes, like and dislike. Lobato and Garza (2017) developed a classification algorithm using neural networks and EEG signals to measure the affective states of “do like” and “don’t like” during buying processes. Yadava et al. (2017) proposed a predictive model using the HMM classifier to analyze EEG signals to understand consumers’ affective states and purchase preferences toward e-commerce goods, and the model achieved an accuracy of 70.33%. They also tested numerous other models, such as the SVM, RF, and KNN. Interestingly, Kumar et al. (2019) creatively put forward a multimodal rating prediction method by fusing the affective ratings from e-commerce websites and EEG data. The ABC and RF models were applied to optimize and compute the ratings from varied data sources, and the results showed that the combined method could achieve a lower Root Mean Square Error (RMSE) in rating prediction compared to a unimodal method. More recently, Aldayel et al. (2020) adopted a deep learning approach to assess consumer preferences (pleasant or unpleasant) by extracting the PSD and valence features. They built four different classifiers, namely, the DNN, RF, SVM, and KNN, which attained accuracies of 94, 92, 62, and 88%, respectively.

In summary, due to significant differences between the experimental design and the paradigm used in these studies, it is difficult to make direct comparisons of the classification accuracies achieved. Furthermore, it seems that there is no particular feature extractor or affective classifier that appears to be the single best choice for all marketing scenarios. In most situations, one should consider as many algorithms as possible from the studies mentioned above and then compare the results with a range of features and algorithms before choosing the one with the best performance for the given marketing application.

## Discussion

In this review, we summarize previous studies analyzing EEG signals as biological markers in affective mechanism and recognition in the marketing area (as shown in Table 1). The majority of the studies, especially those using machine learning techniques and algorithms, have been published in the last 10 years. This review provides new directions regarding neuromarketing data analyses and fosters cooperation among scholars from miscellaneous disciplines, such as information science, neuroscience, marketing, and psychology. Although there has been a recent increase in the number of EEG-based AC studies in marketing with no signs of slowing down, theoretical and operational challenges must be settled before moving forward.

**TABLE 1:** Summary of current findings on EEG-based affective computing in marketing.

Reference	Journal	Marketing substance	Affective states	EEG features	Method (classification accuracy)
Reeves et al., 1989	Human Communication Research	TV commercials	Valence	Hemispheric differences (alpha)	ANOVA
Ma et al., 2007	Neuroreport	Brand	Conflict	ERPs (N270)	ANOVA
Ohme et al., 2009	Journal of Neuroscience, Psychology, and Economics	TV commercials	Valence	Hemispheric differences (alpha)	*t* tests and Pearson’s linear correlation
Chen et al., 2010	Biological Psychology	E-commerce products	Valence	ERPs (N500)	ANOVA
Handy et al., 2010	Journal of Cognitive Neuroscience	Commercial logos	Liking	ERPs (P1, N2)	ANOVA
Ohme et al., 2010	Journal of Economic Psychology	TV Commercials	Valence	Hemispheric differences (alpha)	ANOVA and *post hoc* tests
Vecchiato et al., 2010	Brain Topography	TV commercials	Pleasantness	GFP (theta, beta)	ANOVA
Vecchiato et al., 2011b	Medical,Biological Engineering and Computing	TV commercials	Pleasantness	PSD, ERD (alpha, theta)	*t*-test
Jones et al., 2012	Biological Psychology	Pricing	Anxiety	ERPs (FN400, P3, LPC)	ANOVA
Guo and Elgendi, 2013	Journal of Advanced Management Science	Recommender system for e-commerce	Valence	Spectral power (alpha, beta)	Pearson’s linear correlation
Khushaba et al., 2013	Expert Systems with Applications	Food property	Liking	PSD, ERS (delta, theta, alpha, beta, gamma)	Phase locking value
Lin et al., 2014	Frontiers in Neuroscience	Music	Valence, arousal	PSD, DLAT, DCAU, MESH (delta, theta, alpha, beta, gamma)	SVM (valence: 82.5%; arousal: 79.1%)
Kuan et al., 2014	Journal of Management Information Systems	Group-buying information	Valence, liking	Hemispheric differences (alpha)	ANOVA
Vecchiato et al., 2014	Cognitive Computation	TV commercials	Valence, arousal	PSD (alpha), IAF (alpha)	*t* test
Friedman et al., 2015	International Conference on Affective Computing and Intelligent Interaction	TV commercials	Valence	Spectral power hemispheric differences (delta, theta, alpha, low beta, high beta)	MANOVA, SVM (77.3%), LMT (81.2%)
Pozharliev et al., 2015	Journal of Marketing Research	Luxury goods	Arousal	ERPs (P2, P3, LPP)	ANOVA
Telpaz et al., 2015	Journal of Marketing Research	Consumer goods	Liking	ERPs (N200), spectral power (theta)	*t* tests and spearman correlation
Venkatraman et al., 2015	Journal of Marketing Research	TV commercials	Valence, arousal	Occipital activity and frontal asymmetry (alpha)	SUR regression
Yang et al., 2015	Journal of Physiological Anthropology	TV commercials	Happiness, surprise	PSD (delta, theta, alpha, low beta, high beta, gamma)	ANOVA, FLDA (happiness: 88.6%; surprise: 87.5%)
Berčík et al., 2016a	Periodica Polytechnica Social and Management Sciences	Music preferences	Pleasantness	Spectral power (alpha, beta)	Descriptive statistics
Berčík et al., 2016b	Appetite	Store illumination	Valence, arousal, dominance	Spectral power (alpha, beta)	Non-parametric Wilcoxon signed rank test
Chew et al., 2016	Cognitive Neurodynamics	Industrial design	Liking	ERS/ERD (alpha, theta, delta)	SVM (79%), KNN (80%)
Gupta and Falk, 2016	Neurocomputing	Music videos	Valence, arousal, dominance, liking	EEG graph-theoretic features	SVM (valence: 64%; arousal: 64%; dominance: 59%; liking: 64%), RVM (valence: 65%; arousal: 68%; dominance: 63%; liking: 67%)
Horska et al., 2016	Agricultural Economics	Consumer preferences	Valence	Wave fluctuating tendency	Kruskal–Wallis test
Lee, 2016	Journal of Business Research	Willingness to pay	Valence	Spectral power (theta)	sLORETA
Gauba et al., 2017	Neural Networks	TV commercials	Valence	Statistical mean of band oscillations of each electrode	RF (68%)
Guixeres et al., 2017	Frontiers in Psychology	Online commercials	Liking	GFP (delta, theta, alpha, beta, Gamma)	ANN (82.9%)
Lobato and Garza, 2017	IEEE Latin America Transactions	Purchasing behaviors	Liking	Hemispheric differences (alpha)	ANN (76%)
Yadava et al., 2017	Multimedia Tools and Applications	E-commerce products	Liking	Band oscillations (delta, theta, alpha, beta, Gamma)	HMM (70.3%)
Avinash et al., 2018	Procedia Computer Science	Advertisement jingles	Valence	Frontal asymmetry (theta)	KNN (100%), FLDA (90%)
Jin et al., 2018	Frontiers in Human Neuroscience	Eco-labeled products	Valence	ERPs (P2, N2)	ANOVA
Wei et al., 2018	Frontiers in Neuroscience	Commercials	Valence	Wavelength, signal quality (delta, theta, low alpha, high alpha, low beta, high beta, low gamma, high gamma)	SVM (77.3%)
Kumar et al., 2019	Information Fusion	E-commerce products	Valence	Spectral power (delta, theta, alpha, beta, Gamma)	RF (48%), ABC + RF (72%)
Aldayel et al., 2020	Applied Sciences	Purchasing behaviors	Pleasantness	PSD (theta, alpha, beta, gamma)	DNN (94%), RF (92%), SVM (62%), KNN (88%)
Shang et al., 2020	Psychology Research and Behavior Management	Webpage layout	Valence	ERPs (P2, LPP)	ANOVA

*ANOVA, analysis of variance; ERPs, event-related potentials; GFP, global field power; PSD, power spectral density; SVM, support vector machine; IAF, individual alpha frequency; MANOVA, a multivariate analysis of variance; LMT, logistic model tree; FLDA, Fisher linear discriminant analysis; ERS/ERD, event-related synchronization/desynchronization; KNN, K-nearest neighbors; RVM, relevance vector machine; sLORETA, standardized low-resolution electromagnetic tomography; RF, random forest; ANN, artificial neural network; HMM, hidden Markov model; ABC, artificial bee colony; DNN, deep neural network.*

First, it would be helpful to pay more attention to multiclass affective classification. As this review shows, most of the previous studies are based on dimensional emotion theory, typically concerning the dimensions of arousal, valence, liking, and dominance. The state of the art usually relies on the affective polarity of its components (e.g., positive or negative) and proposes approaches that mostly focus on binary affective classification. However, to study the affective states of consumers, it would be more interesting to go deeper into the classification and detect subtle affective changes in marketing. Furthermore, marketing scenarios may induce multiple emotions in customers. The phenomena of coexistence should be considered in affective tagging. We recommend that future studies should focus on two issues to develop a more accurate affective definition and conduct better forecasting: (1) they should aim for a deeper understanding of consumer ambivalence, characterized by the co-occurrence of positive and negative emotions (Kreibig and Gross, 2017; Hu et al., 2019); and (2) consider emotion dyads, namely, a mix of primary emotions, raised by Plutchik (1980).

The multidimensional and multimodal feature fusion can obtain better recognition performance. When studying EEG-affect relationships, EEG-based AC studies assume that EEG signals can sufficiently depict and predict human affective states. However, this hypothesis cannot always be assumed to be true because the relationship between physiological responses and psychological states could be very complex (Cacioppo and Tassinary, 1990; Hu et al., 2019). To achieve precise prediction and improved generalization, first, we suggest decreasing the abundant number of features from EEG signals and further perform feature selection and fusion. The most widely used features include differential symmetry, GFP, PSD, and ERPs. It might be the case that a fusion of features derived from different EEG signal types will lead to better recognition performance (Hakim and Levy, 2019). It is worth noting that future studies should be more cautious regarding the reliability and validity of “one-to-one” relationships (one affective state is associated with one and only one EEG feature) (Bridwell et al., 2018; Hu et al., 2019). Second, recent studies have revealed that multimodal frameworks can effectively increase emotion recognition accuracy and robustness compared to unimodal frameworks (Guixeres et al., 2017; Avinash et al., 2018; Kumar et al., 2019). The advantage of multiple modalities (for example, vision, sound, or smell) helps to increase the validity and usability since the weaknesses of one modality are offset by the strengths of another. Future studies may derive features from modalities other than EEG while collecting and analyzing data by using machine learning, natural language processing, and automatic speech recognition technology, evolving from unimodal analyses to multimodal fusion.

The use of portable wireless EEG devices and virtual reality (VR) technology can alleviate the lack of ecological validity in marketing studies. For EEG hardware devices, the whole-brain coverage, the time-consuming preparation procedure, and the prohibitive cost of a professional headset with wet electrodes make it impractical and difficult to transfer the laboratory to real-world applications in marketing. A group of recent studies has confirmed the feasibility of using consumer-level EEG headsets for AC with promising results. For example, the widely used wireless EPOC headset (e.g., Kuan et al., 2014; Lin et al., 2014; Friedman et al., 2015; Yang et al., 2015; Gauba et al., 2017; Yadava et al., 2017; Kumar et al., 2019), due to its light weight, low price, and ease of use, shows promise. Studies on the EPOC headset seem to agree that it can be applied to acquire reliable EEG signals in marketing, but researchers should pay attention to its relatively low signal-to-noise ratio and poor signal stability (Friedman et al., 2015). We suggest that researchers evaluate the performance of consumer-level devices using the standard testing procedures proposed by Hu et al. (2019). In addition, to bridge the gap between the laboratory environment and real market scenarios, the use of VR is an important trend that can effectively enhance the experience of immersive sensation. It enables consumers to get a direct, intuitive, and concrete understanding of the appearance, quality, and performance of products (Guo and Elgendi, 2013). Furthermore, VR makes it possible to simulate and assess retail and consumption environments under controlled laboratory conditions (Marín-Morales et al., 2017), allowing the isolation and modification of variables in a cost-effective manner.

Studying interactions among multiple customers is critical for understanding the marketing ecosystem, which consists of interrelated trends that shape consumer behaviors. Most AC studies in marketing have concentrated mainly on a single consumer’s EEG activity and may ignore the socio-affective interaction and processes related to consumer behavior (Hasson et al., 2012). The EEG-based hyperscanning technique [for a recent review, see Liu et al. (2018)] provides a way to explore dynamic brain activities between two or more interactive customers and their underlying neural affective mechanisms. In previous hyperscanning studies, interpersonal neural synchronization (INS) has been verified to be a crucial neural marker for different kinds of social interactions, such as communication (Stephens et al., 2010), collaborative decision making (Montague et al., 2002; Hu et al., 2018), and imitation (Pan et al., 2017). As consumer behavior is inherently social and interactive in nature, EEG-based INS could be used to study the biological mechanism for shared intentionality of consumption, panic buying, collective emotion, and group purchase.

## Author Contributions

Both authors listed have made a substantial, direct, and intellectual contribution to the work and approved it for publication.

## Conflict of Interest

The authors declare that the research was conducted in the absence of any commercial or financial relationships that could be construed as a potential conflict of interest. The handling editor declared a shared affiliation with TL and GP at the time of review.
